# The role of nasal microbiota and type 2 innate lymphoid cells in the pathogenesis of allergic rhinitis

**DOI:** 10.1016/j.bbrep.2026.102602

**Published:** 2026-05-04

**Authors:** Chen Wang, Yi-Ming Zhang, Min-Li Zhou, Min Li, Ke-Jia Cheng

**Affiliations:** Department of Otolaryngology, The First Affiliated Hospital, School of Medicine, Zhejiang University, Hangzhou, China

**Keywords:** Allergic rhinitis, ILC2s, Type 2 cytokines, 16S rRNA, Autophagy, Mitophagy

## Abstract

Allergic rhinitis (AR) is a type 2 inflammation-related disease, potentially associated with innate lymphoid cells (ILC2s), nasal microbiota, and autophagy. Mice were divided into control, AR, AR + IL-33, and AR + antibiotic groups(n = 5). ELISA was used to measure IL-4, IL-5, and IL-13, Masson staining to evaluate tissue remodeling, flow cytometry to detect ILC2s and memory ILC2s, 16S rRNA sequencing to analyze nasal microbiota, and Western blot to assess autophagy and mitophagy proteins. Compared with controls, mice in each AR group exhibited more nasal symptoms, enhanced tissue remodeling, and altered microbiota diversity with reduced Proteobacteria and increased Firmicutes. IL-33 further elevated type 2 cytokines in serum and nasal lavage fluid, increased nasal ILC2s and miR-155 expression, but did not affect memory ILC2s. All treatment groups showed increased p62 and LC3II/LC3I ratio, along with decreased FUNDC1 and BNIP3L levels. These findings suggest that AR is characterized by type 2 inflammation, tissue remodeling, and microbial dysbiosis, with IL-33 aggravation. Autophagy and mitophagy dysfunction may contribute to AR pathogenesis.

## Highlights

• AR shows type 2 inflammation and tissue remodeling, aggravated by IL-33.
• Elevated ILC2s and miR-155 upregulation drive type 2 inflammation.
• Nasal dysbiosis: IL-33 group shows reduced Proteobacteria and increased Firmicutes.
• Impaired autophagy and mitophagy may contribute to AR pathogenesis.

## 1 Introduction

Allergic rhinitis (AR) is characterized by eosinophilic inflammation resulting from sensitization to seasonal or perennial allergens [1]. However, the pathogenesis of this disease remains unclear, and its suboptimal therapeutic efficacy and recurrent episodes are linked to uncontrolled eosinophilic inflammation and progressive nasal mucosa remodeling [2].

Type 2 innate lymphoid cells (ILC2s) constitute one of the subgroup of innate lymphoid cells (ILCs), with functions analogous to T helper (Th) 2 cells [3]. Allergen exposure triggers epithelial cells to release signals that activate ILC2s, leading to the production of type 2 inflammatory cytokines, including interleukin (IL)-4、IL-5 and IL-13 [4]. ILC2s play a pivotal role in the development and maintenance of airway allergic inflammation and tissue remodeling processes [5]. Studies show that AR mice have higher ILC2s levels than controls, leading to increased IL-5 and IL-13 production [6]. The microRNA-155 (miR-155)/GATA3 axis critically regulates ILC2s function and type 2 cytokine production in AR models [7]. Clinically, AR patients exhibit elevated miR-155 expression in ILC2s [8]. Therefore, ILC2s may represent a promising new therapeutic target for the management of AR.

Traditionally, immune memory has been regarded as a hallmark of adaptive immunity, referring to the memory effect of the immune system on specific antigens that have been previously encountered, leading to an enhanced and stronger immune response when the same pathogen is subsequently exposed [9]. However, recent studies have shown that innate immune cells can also develop immune memory. After a transient exposure to pathogens or endogenous stimuli, innate immune cells undergo epigenetic and metabolic reprogramming, leading to a long-lasting state of enhanced functional responsiveness. Upon secondary stimulation, they exhibit amplified effector responses—a process referred to as trained immunity [10,11]. In particular, IL-33 re-stimulation has been reported to elicit enhanced IL-5 and IL-13 production, prolonged survival, and augmented effector function in IL-33–experienced ILC2s [12]. IL-33 signaling also promotes ILC2 effector programs and is associated with increased expression of molecules such as PPARγ and its downstream target ST2 [13]. In female BALB/c mice, IL-33-experienced ILC2s exhibit higher ST2 expression and enhanced secondary responsiveness [14]. Following in vivo IL-33 administration in mice, ILC2s in bronchoalveolar lavage fluid exhibit a relatively uniform ST2^+^ICOS^+^ phenotype, indicative of a highly activated and phenotypically consistent ILC2 population [15]. In mouse lung models, an ICOS^+^ST2^+^ILC2 population has been described to display recall-like responses and to promote eosinophil infiltration and type 2 inflammation [16]. Therefore, in the present study, we analyzed ICOS^+^ST2^+^ILC2s as a memory-like ILC2 enriched subset, while acknowledging that no consensus marker set has yet been established.

The human nasal mucosa hosts a diverse microbial community, including commensal microorganisms that maintain symbiotic relationships with the host [17]. Reduced bacterial diversity is linked to pathogen dominance and associated with allergic diseases like atopic dermatitis, AR, and asthma [18]. In patients with AR, the nasal microbiota's abundance and composition differ from healthy individuals, and these differences may worsen AR symptoms [19]. There may be a close interplay between the host microbiota and ILCs [20].However, the role of nasal microbiota contribution to AR pathogenesis remains controversial. Notably, no studies to date have investigated the interaction between nasal microbiota and ILC2s in AR.

Autophagy helps maintain cellular homeostasis by removing misfolded proteins and defective or excessive organelles [21]. Mitophagy is the process of degrading damaged mitochondria. Autophagy has been implicated in airway allergic inflammations like asthma and AR,aiding tissue remodeling [22]. Elevated autophagy levels have been observed in the nasal mucosa of both AR patients and mouse models [23]. Notably, recent studies reveal that autophagy critically regulates the survival and effector functions of ILC2s [24].

Therefore, in this study, the AR mouse model was established via intraperitoneal injection and nasal sensitization of ovalbumin (OVA). Furthermore, in the IL-33 group, ILC2s were activated through IL-33 stimulation, while in the antibacterial group, the nasal flora structure was artificially modulated by administering multiple antibiotics via nasal drops. We measured type 2 nasal inflammation, tissue remodeling, levels of ILC2s and memory-like ILC2s, nasal microbiota diversity, as well as autophagy and mitophagy levels in each group of mice. The impact of ILC2s activation on the nasal microbiota was observed by promoting ILC2s activation with the addition of IL-33. This study aims to preliminarily investigate the roles of nasal microbiota and ILC2s in the pathogenesis of AR.

## 2 Methods

### 2.1 Animal models

Female Balb/c mice (4-6 weeks old) were purchased from Hangzhou Medical College (Hangzhou, Zhejiang, China). Mice were housed in a specific-pathogen-free isolation environment in the Animal Experimental Center of The First Affiliated Hospital of Medical School of Zhejiang University (licence no. SYXK(Zhe)2023-0021). The mice were housed at room temperature with a 12 h light/dark cycle and a moderate humidity(50–60%),with free access to food and water. The experiments described herein were approved by the Ethics Committee of the First Affiliated Hospital, School of Medicine, Zhejiang University (licence no:2019933).

Mice were randomly divided into four groups(n = 5): the control group, the AR group, the IL-33-treated AR group, and the antibiotic-treated AR group. AR mice were intraperitoneally injected with OVA solution (50 μg OVA (A5503; Sigma-Aldrich, USA) and 1 mg aluminium hydroxide (Sigma-Aldrich, USA)/200 μl PBS)on day 0、7 and 14. From day 21 to 27, OVA (400μg/20μlPBS) was administered via intranasal instillation without anesthetized. Mice in the IL-33+AR group were given 0.25μg/mouse rmIL-33 nasal drops for three consecutive days from the day 21to day 23. We pretreated mice with a broad-spectrum antibiotic cocktail to induce nasal microbiota depletion prior to sensitization. In the antibiotic + AR group, mice were first given a mixed antibiotic intranasal drip daily (vancomycin (1 mg/kg/day) + ampicillin (100 mg/kg/day) + kanamycin (100 mg/kg/day) + metronidazole (0.2 mg/kg/day)), and OVA sensitization began after a total of 2 weeks. Control group mice received intraperitoneal injection and nasal drops of the same volume of phosphate buffered saline (PBS) as the AR group. (Fig. 1A). After the final intranasal challenge, mice were individually placed in a clean observation cage. The frequency of nose scratches and sneezes was recorded for 10 min. A sneeze was defined as a sudden, forceful expiration through the nose, and a nasal scratch was defined as a rapid pawing/rubbing movement directed toward the nasal region. Behavioral scoring was performed by an observer blinded to group allocation.

**Fig. 1:**
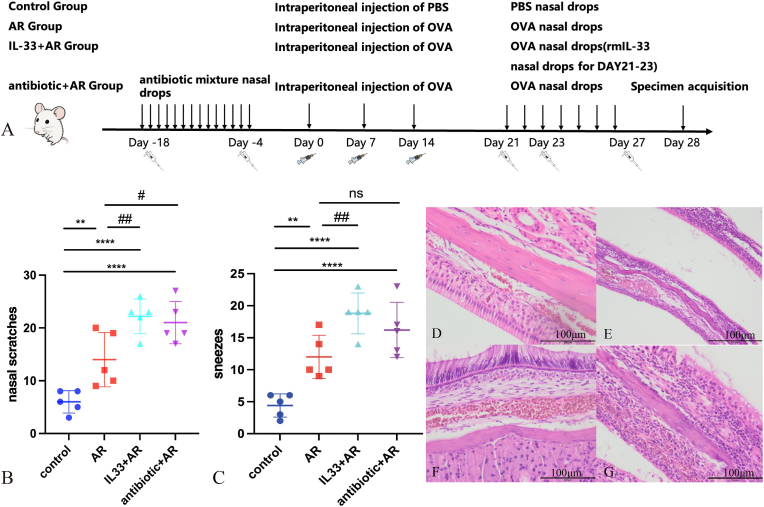
Mice modeling methods and behavioral experimental results. A: Method diagrams for establishing mouse models in each group: the control group, the AR group, the IL-33-treated AR group, and the antibiotic-treated AR group. B: The frequency of nose scratching within 10 min after the l final challenge of mice in each group.C: The frequency of sneezing within 10 min after the final challenge of mice in each group; D:Microscopic image of HE stained nasal mucosa from control group mice (400 × magnification); E: Microscopic image of HE stained nasal mucosa from AR group mice (400 × magnification); F: Microscopic image of HE stained nasal mucosa from IL-33+ AR group mice (400 × magnification); G: Microscopic image of HE stained nasal mucosa from antibiotic + AR group mice (400 × magnification). Compared with the control group, ∗∗P < 0.01, ∗∗∗∗P < 0.0001; compared with the AR group, #P < 0.05, ##P < 0.01, ns: No statistically significant difference.

### 2.2 Hematoxylin-eosinstaining (HE) staining

the nasal specimens were fixed, then decalcified in decalcification solution (Solarbio, Beijing, China) for another 24 h. Tissues were then dehydrated, embedded, and sectioned vertically. The sections were dewaxed, rehydrated, stained with hematoxylin, counterstained with eosin after hydration, and dehydrated again. Histological features were examined under a microscope at 400 × magnification.

### 2.3 Masson staining

For Masson staining, tissue sections were dewaxed, rehydrated, and stained with a series of dyes including Wright's hematoxylin, lichen red, and aniline blue. The specific operation steps were followed by the manufacturer's direction (Solarbio, Beijing, China). Three random fields were imaged at 400 × magnification. Collagen fibers appeared blue or green, and nuclei dark blue. Blue-stained areas were measured using Image J.

### 2.4 Enzyme-linked immunosorbent assay (ELISA)

Retro-orbital blood was collected using a heparinized capillary tube 24 h after the final challenge. After resting for 30 min, the samples were centrifuged at 3000 rpm for 10 min to collect the serum. Use a blunt-tipped needle inserted through the larynx into the trachea to reach the posterior nasal cavity, infuse 500 μL PBS, perform the lavage twice, and collect the nasal lavage fluid (NLF) from the anterior nostrils. IL-4, IL-5, and IL-13 were measured using paired antibody quantitative enzyme-linked immunosorbent assays (ELISA) following the manufacturer's directions (Elabscience, Wuhan, China).

### 2.5 RT-qPCR

Total RNA was extracted from the nasal mucosa of each group of mice using Trizol (Invitrogen, California, USA) and finally dissolved in diethyl pyrocarbonate (DEPC)-treated water. cDNA was synthesized via reverse transcription using the stem-loop reverse transcription kit (Vazyme, Nanjing, China). qPCR was performed using a Vazyme kit, and Ct values were normalized to U6. Relative expression was calculated using the 2-ΔΔCt method. The primers used in this study are as follows:

U6: reverse transcription sequence: 5′-AAAATATGGAACGCTTCACGAATTTGC-3’;

qPCR forward: 5′CCTGCTTCGGCAGCACA-3’;

qPCR reverse:5′-TGGAACGCTTCACGAA-3’;

miRNA155: reverse transcription sequence:5‘-GTCGTATCCAGTGCAGGG-TCCGAGGTATTCGCACTGGATACGACCTGTTAAT-3’;

qPCR forward:5′- GTGCCTCCAACTGACTCCTACA-3’;

qPCR reverse:5′-GTCGTATCCAGTGCAGGGTC-3’.

### 2.6 Cell isolation and flow cytometry

Mouse nasal mucosa was cut into 1–2 mm^3^ pieces and digested in 1 ml of RPMI 1640 containing 1 mg/ml collagenase IV and dispase II at 37 °C for 90 min with shaking. The cells were split into two tubes with 500 μl of complete RPMI 1640 resuspend. For ILC2 detection, the first tube was stained with PerCP-CD45, FITC-lineage, APC-CD90.2, and PE-KLRG1 antibodies (1:200, BioLegend, USA). For memory-like ILC2s, the second tube included these same antibodies plus BV421-ST2 and BV785–ICOS (1:200, BioLegend, USA). Both tubes were incubated at room temperature in the dark for 25 min. Flow cytometry showed that ILC2s were Lin^−^CD45^+^CD90.2^+^KLRG1^+^ cells. We operationally defined putative memory-like ILC2s as ICOS^+^ST2^+^ subset within the Lin^−^CD45^+^CD90.2^+^KLRG1^+^ ILC2 gate, based on prior reports describing allergen–experienced ILC2 populations with memory-like recall features [16].

### 2.7 16S ribosomal RNA gene sequencing (16S rRNA) for microbial biodiversity analysis

After removing the skin over the nasal cavity, the cavity was split along the nasal septum. Sterile swabs were used to gently collect samples from the nasal septum and lateral wall mucosa, avoiding any blood contamination. The samples were then sent to Shanghai Meiji Company for 16S rRNA biodiversity analysis.

Taxonomic assignment of ASVs was performed using the Naive bayes consensus taxonomy classifier implemented in Qiime2 and the SILVA 16S rRNA database (v138). Bioinformatic analysis of the nasal microbiota was carried out using the Majorbio Cloud platform (https://cloud.majorbio.com). Alpha diversity (Shannon index) was calculated using Mothur v1.30.1, and beta diversity was assessed using principal coordinate analysis (PCoA) based on Bray-Curtis dissimilarity with the Vegan v2.5-3 package. Differences in bacterial groups between sample groups were identified using LEfSe (http://huttenhower.sph.harvard.edu/LEfSe), with significant taxa defined by LDA score >2 and P < 0.05.

### 2.8 Western blot

Equal amounts of protein (30 μg) were separated by SDS-PAGE, then transferred to PVDF membranes and blocked. The membranes were incubated overnight at 4 °C with primary antibodies, including β-actin (1:50000, 66009-1-Ig,ProteinTech, Wuhan,China), p62(1:2000, 5114,Cell Signaling Technology, US), BECLIN1(1:2000,11306-1-AP,ProteinTech, Wuhan,China), LC3(1:1000,12741,Cell Signaling Technology,USA), FUNDC1(1:8000, bs-13227R,Bioss, Beijing,China), BNIP3L(1:1000, 12986-1-AP ProteinTech, Wuhan,China), PINK1(1:1000,23274-1-AP,ProteinTech, Wuhan,China),and Parkin(1:1000, 14060-1-AP,ProteinTech, Wuhan, China). After color development, proteins were visualized with a BIO-RAD ChemiDoc system.

### 2.9 Statistical analysis

Statistical analysis was performed using SPSS (version 26.0). Data with normal distribution were shown as mean ± SD and analyzed by one-way ANOVA. Non-normal data were shown as median (Interquartile Range, IQR) and analyzed using the Kruskal-Wallis test. A p-value <0.05 was considered statistically significant. Graphs were created using GraphPad Prism (version 8.3.0).

## 3 Results

### 3.1 Establishment of an allergic rhinitis–like phenotype in mice

In this study, we quantified the frequency of sneezing and nasal scratching in mice within 10 min after the final challenge to evaluate the degree of sensitization. Compared to the control group, the AR, IL-33+AR, and antibiotic + AR groups showed more sneezing and scratching, with statistically significant differences. The IL-33+AR group had significantly higher counts than the AR group.(Fig. 1B–C). Collectively, these behavioral readouts indicate successful induction of an AR-like symptomatic phenotype in the OVA-challenged mice.

### 3.2 The levels of type 2 inflammation and tissue remodeling of each AR treatment group increased

#### 3.2.1 HE staining

The HE staining results of the nasal mucosa of each group were examined under a microscope at 400 × magnification. The control group maintained an intact and well-organized pseudostratified epithelium with minimal inflammatory cell infiltration. In contrast, the AR group, the IL-33+AR group, and the antibiotic + AR group exhibited increased inflammatory cell infiltration in the submucosa, accompanied by mild epithelial irregularity and stromal edema、congestion(Fig. 1D–G).

#### 3.2.2 The level of type 2 inflammation was elevated in AR treated groups

**Fig. 2:**
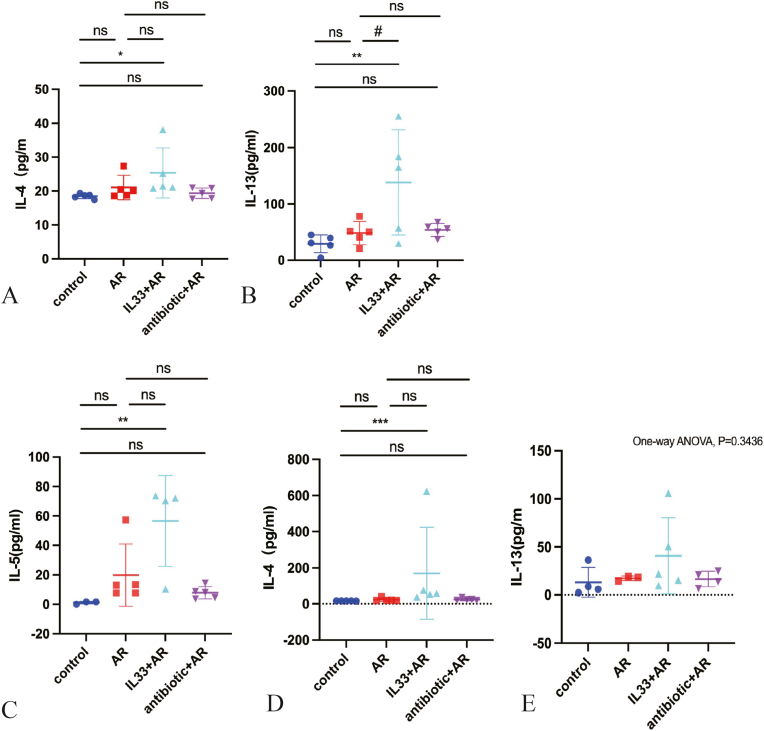
The levels of type 2 inflammatory factors in the peripheral blood and NLF of mice in each group were detected by ELISA A、B: Comparison of the levels of type 2 inflammatory cytokines IL-4 and IL-13 in the serum across all groups; C、D、E: Comparison of the levels of type 2 inflammatory cytokines IL-4、IL-5 and IL-13 in the nasal lavage fluid across all groups. Compared with the control group, ∗P < 0.05, ∗∗P < 0.01, ∗∗∗P < 0.0001. Compared with the AR group, #P < 0.05, ns: No statistically significant difference.

Next, we utilized the ELISA method to quantify the levels of type 2 inflammatory cytokines, including IL-4, IL-5, and IL-13, in the serum and NLF of mice across all experimental groups. The IL-33+AR group had higher IL-4 and IL-13 levels than the control group in both serum and NLF, with statistically significant differences. IL-13 was also significantly higher in the IL-33+AR group compared to the AR group in serum samples(Fig. 2).

#### 3.2.3 The nasal tissue remodeling level was elevated in all treated groups

**Fig. 3:**
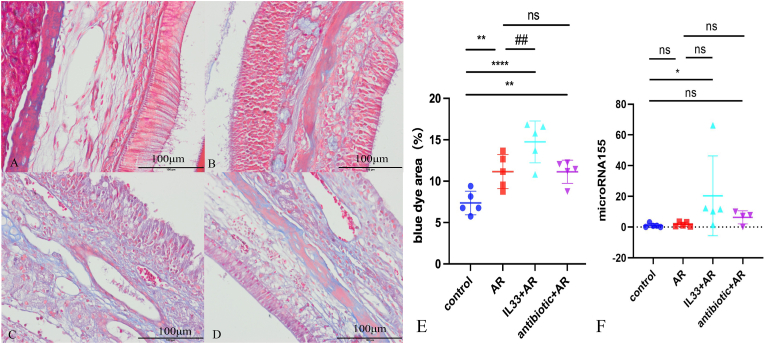
Masson staining was used to observe tissue remodeling level of each group. A: Microscopic image of Masson stained nasal mucosa from control group mice (400 × magnification); B: Microscopic image of Masson stained nasal mucosa from AR group mice (400 × magnification); C: Microscopic image of Masson stained nasal mucosa from IL-33+AR group mice (400 × magnification); D: Microscopic image of Masson stained nasal mucosa from antibiotic + AR group mice (400 × magnification); E: Comparison of the area of collagen blue-dyed nasal mucosa in each group of mice. F: Comparison of the miRNA-155 level of nasal mucosa in each group of mice. Compared with the control group, ∗P < 0.05, ∗∗P < 0.01, ∗∗∗P < 0.0001. Compared with the AR group, ##P < 0.01, ns: No statistically significant difference.

We examined Masson-stained nasal mucosa at 400 × magnification and measured the blue-stained collagen fiber area in three random fields to evaluate the tissue remodeling levels. Compared to the control group, the AR, IL-33+AR, and antibiotic + AR groups showed significantly increased collagen areas. The IL-33+AR group had a notably larger stained area than the AR group, with statistical significance (Fig. 3A–E).

### 3.3 The expression level of miRNA-155 in the IL33+AR group was significantly elevated

MiR-155 is a well-recognized regulator of allergic type 2 inflammation and has been reported to modulate cytokine programs in multiple immune cell types, including innate lymphoid cells. Subsequently, we performed RT-qPCR to detect the expression levels of miRNA-155 in nasal mucosa samples from all groups of mice. Compared with the control group, the expression level of miRNA-155 in the IL-33+AR group exhibited a increase, with statistically significant differences. Although miRNA-155 levels were also higher in the antibiotic + AR group, the differences were not statistically significant (Fig. 3 F).

### 3.4 ILC2s levels in the nasal mucosa were significantly increased in AR and IL33+AR group

**Fig. 4:**
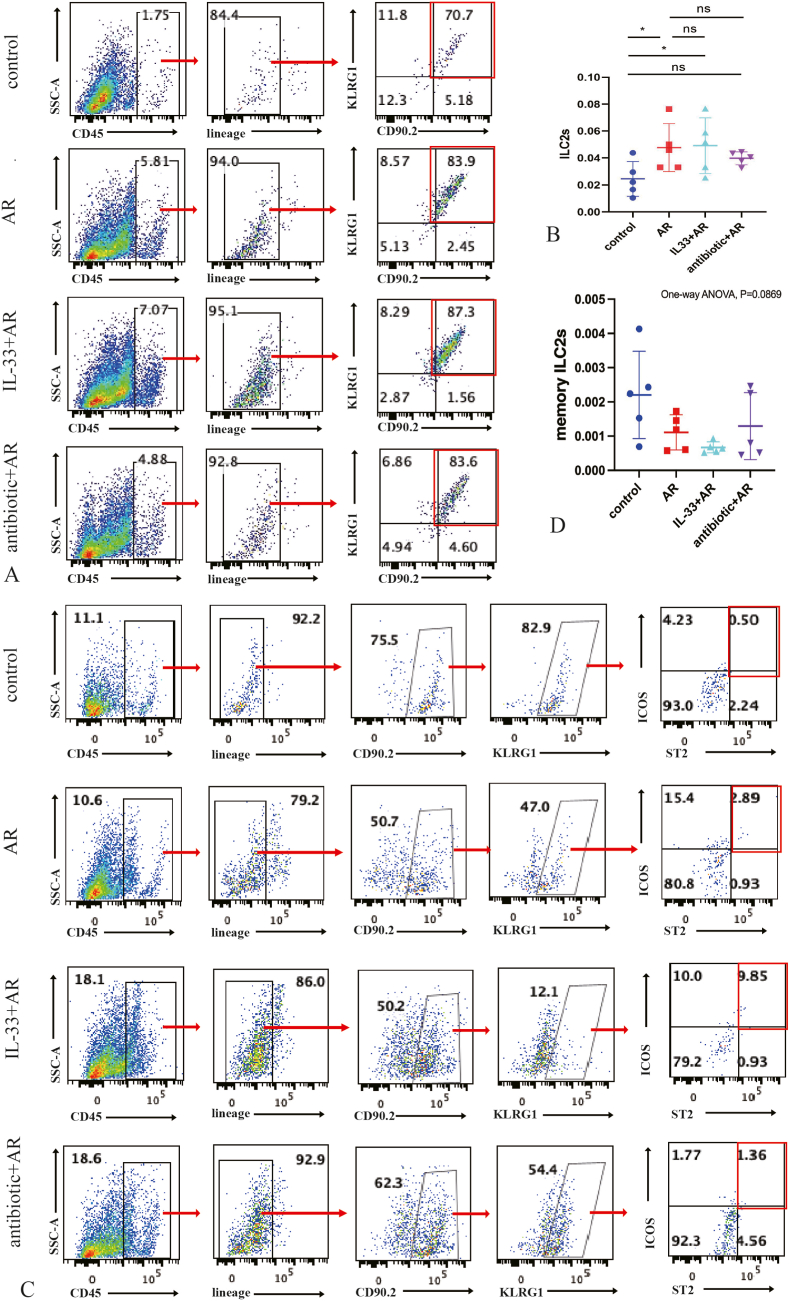
flow cytometry detection of ILC2s and memory ILC2s levels in the nasal mucosa in each group of mice. A: Flow cytometry results of ILC2s levels in the nasal mucosa in each group of mice; B: Comparison of ILC2s levels in nasal mucosa in each group of mice; C: Flow cytometry results of memory ILC2s levels in nasal mucosa in each group of mice; D: Comparison of ILC2s levels of nasal mucosa in each group of mice. Compared with the control group, ∗P < 0.05, ns: No statistically significant difference.

Flow cytometry was employed to quantify ILC2s and memory-like ILC2s levels in the nasal mucosa in each group of mice. ILC2 levels were markedly higher in the AR and IL-33+AR groups compared to the control group, with statistically significant differences. The antibiotic + AR group also showed increased ILC2s, but the difference was not statistically significant (Fig. 4A and B). No significant differences were found in memory ILC2 levels between groups (Fig. 4C and D). The gating strategy is shown in detail in Supplementary Fig. 1.

### 3.5 The nasal microbiota diversity and relative abundance change in mice of treatment groups

**Fig. 5:**
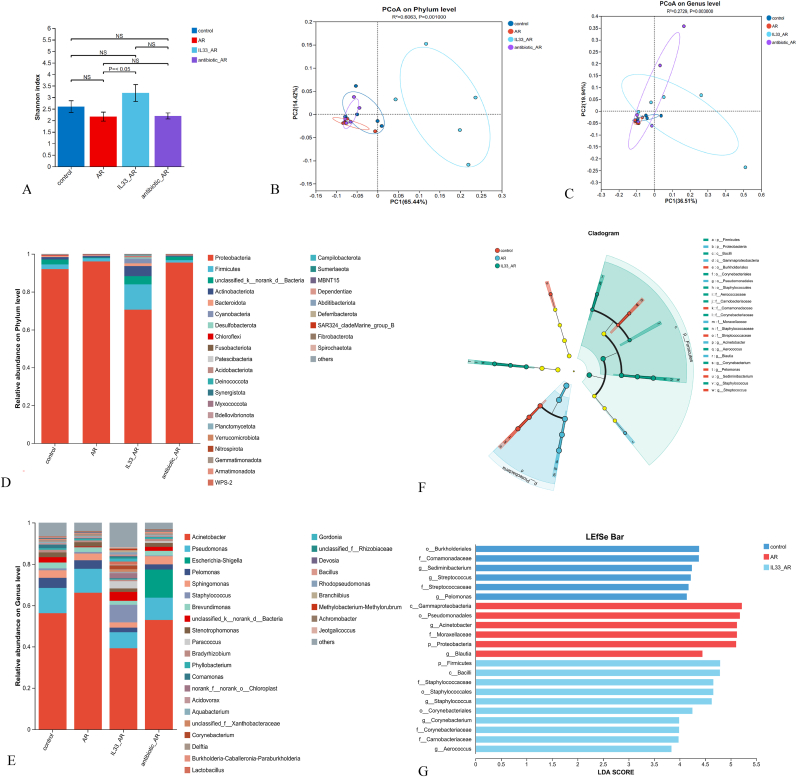
Analysis of the diversity and distribution of nasal microbiota in each group of mice by 16S rRNA biodiversity analysis. A: Simpson values of nasal microbiota in each group; B: PCoA of nasal microbiota composition at the phylum level in each group (Bray–Curtis distance); C: PCoA of nasal microbiota composition at the genus level in each group (Bray–Curtis distance); D: bar plots of nasal microbiota at the portal level in each group; E: bar plots of nasal microbiota at the genus level in each group; F: Multi-level taxonomic cladogram of LEfSe analysis of nasal microbiota in each group; G: LDA histogram of LEfSe analysis of nasal microbiota in each group of mice.

Subsequently, nasal microbial swabs were collected for 16S rRNA biodiversity analysis. All mice originated from the same batch, were raised in identical environmental conditions, and received nasal drops prepared at the same time. A higher Shannon value indicates greater community diversity. The IL-33+AR group showed significantly greater diversity than the AR, surpassing both the control group and the antibiotic + AR group, although no statistically significant differences. In the antibiotic + AR group, alpha diversity was reduced compared with controls, consistent with microbiota depletion caused by broad-spectrum antibiotics. Overall, the IL-33+AR group had the most diverse nasal microbiota (Fig. 5A). In the PCoA plot, closer points indicate more similar communities. At the phylum level, the IL-33+AR group showed distinct separation from the other groups, while the other three groups had partial overlap. At the genus level, all groups showed some overlap (Fig. 5B and C).

We analyzed the colony composition at both the phylum and genus levels. At the phylum level, Proteobacteria were the most abundant in all groups (91.9%, 96.0%, 79.6%, 95.4%), followed by Firmicutes and Actinobacteria. In the IL-33+AR group, Proteobacteria levels were notably lower, while Firmicutes were significantly higher than in other groups. Actinobacteria, Bacteroidetes, and Cyanobacteria were also higher, though not significantly (Fig. 5D). At the genus level, the IL-33+AR group showed lower levels of Acinetobacter, Pseudomonas, Pelomonas and Sphingomonas, compared to the other groups. Conversely, genera such as Staphylococcus, Paracoccus, Corynebacterium, Lactobacillus, Gordonia, and Rhodopseudomonas were more abundant compared to the others. In the antibiotic + AR group, Escherichia-Shigella and Sphingomonas were more prevalent than in the other three groups. In the control group, Mucomonas was observed at higher levels compared to each treatment group (Fig. 5E).

Furthermore, differences were noted in the Burkholderiales order, Pseudomonadaceae family, Streptococcus genus, Streptococcaceae family, and Pseudomonas genus when comparing the control group with each treatment group. Lastly, In the AR group, notable variations were detected in the Proteobacteria class C, Pseudomonas order, Acinetobacter genus, Moraxellaceae family, Proteobacteria phylum, and Brantaria genus when compared to the other groups. Additionally, in the IL-33+AR group, distinct differences were identified in the Firmicutes phylum, Bacilli class, Staphylococcaceae family, Staphylococcus order, Staphylococcus genus, Corynebacterium genus, Corynebacteriaceae family, Clostridiaceae family, and *Streptococcus pneumoniae* genus compared to the other three groups (Fig. 5F and G).

### 3.6 The mitochondrial autophagy levels decrease in each treatment group

Finally, we employed WB to evaluate the levels of autophagy-related proteins p62, BECLIN1, and LC3II/LC3I, as well as mitochondrial autophagy-related proteins FUNDC1, BNIP3l, PINK1 and parkin in the nasal mucosa in all groups of mice.

**Fig. 6:**
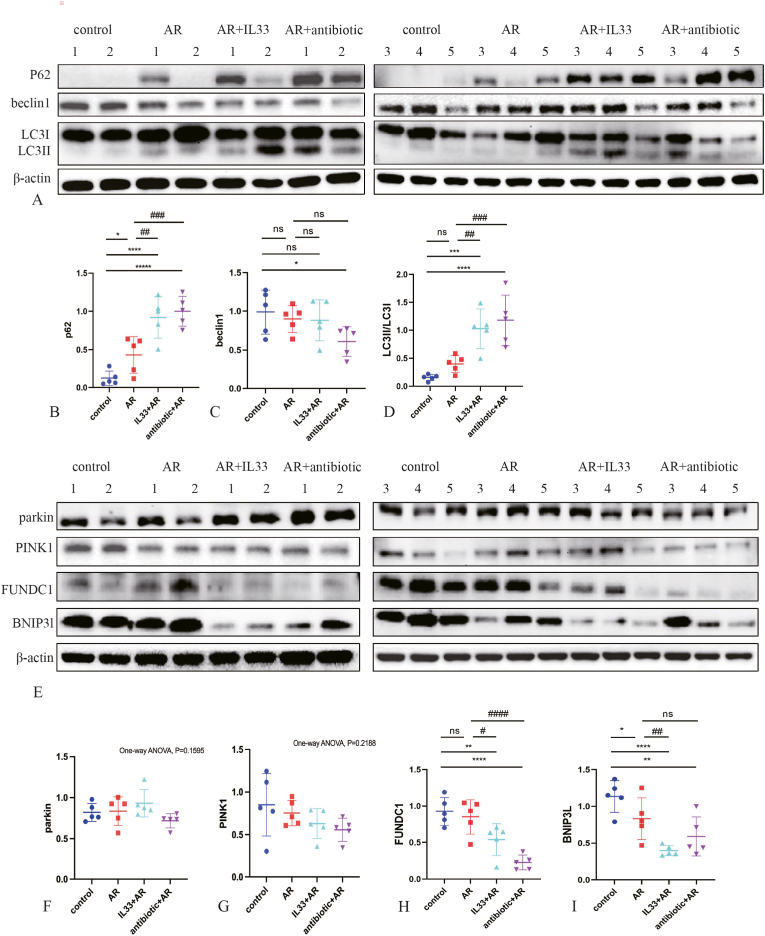
Western blot analysis of autophagy and mitophagy-related protein levels in nasal mucosa of mice from each group. A: Representative WB gels of autophagy-related proteins p62, BECLIN1 and LC3II/LC3I in nasal mucosa across all groups. B-D: Comparison of p62、beclin1 and LC3II/LC3I ratio levels in nasal mucosa in each group of mice (normalized to β-actin).E: Representative WB gels of mitophagy-related proteins parkin、PINK1、FUNDC1 and BNIP3l levels in nasal mucosa across all groups.F–I: Comparison of parkin、PINK1、FUNDC1 and BNIP3l protein levels in nasal mucosa in each group of mice(normalized to β-actin). Compared with the control group, ∗P < 0.05, ∗∗P < 0.01, ∗∗∗P < 0.001, ∗∗∗∗P < 0.0001. Compared with the AR group, #P < 0.05,##P < 0.01, ###P < 0.001, ####P < 0.0001. ns: No statistically significant difference.

Compared to the control group, p62 levels were significantly higher in the AR, IL-33+AR, and antibiotic + AR groups. p62 was also significantly higher in the IL-33+AR and antibiotic + AR groups compared to the AR group, with statistically significant differences (Fig. 6A and B). BECLIN1 levels were significantly lower in the antibiotic + AR group compared to controls, with no significant differences between the AR and IL-33+AR groups (Fig. 6A–C). The LC3II/LC3I ratio was significantly increased in the IL-33+AR and antibiotic + AR groups compared to the control and AR groups. The AR group also showed a higher ratio than controls, but without statistical significance (Fig. 6A–D).

There were no significant differences in Parkin and PINK1 protein levels among all groups (Fig. 6E–G). FUNDC1 levels were significantly reduced in the IL-33+AR and antibiotic + AR groups compared to both the control and AR groups, with statistically significant differences. The AR group also showed a decrease, but it was not significant (Fig. 6E–H). BNIP3L levels were significantly lower in the AR, IL-33+AR, and antibiotic + AR groups than in the control group. Additionally, the IL-33+AR group had significantly lower BNIP3L levels than the AR group. The differences were all statistically significant (Fig. 6E–I).

## 4 Discussion

Type 2 inflammation is marked by increased levels of cytokines like IL-4, IL-5, and IL-13, and damage to barrier function [25]. In this study, Mice in each treatment group showed more obvious symptoms of AR such as nasal scratching and sneezing, indicating successful induction of an AR-like symptomatic phenotype. The levels of type 2 inflammation in mice of each AR treatment group increased, which was consistent with the literature reports. Notably, the level of type 2 inflammation and AR symptoms in the IL-33+AR group was greater than that observed in the AR group. Microscopic examination of HE-stained sections revealed that, all AR groups had damaged and disorganized nasal epithelial barriers compared to the control group. Epithelial dysfunction in AR may facilitate easier penetration of allergens into the submucosa, thereby triggering a cascade of allergic reactions [26].

Tissue remodeling is a common feature of respiratory allergic diseases like asthma and AR. It involves epithelial damage, increased fibroblast activity, muscle thickening, and vascular remodeling, all of which contribute to heightened bronchial hyperresponsiveness [27].In our study, remodeling was significantly increased in all AR treatment groups compared to controls. The IL-33+AR group showed the highest level. These findings suggest that nasal tissue remodeling may be enhanced in AR and could be associated with the magnitude of local type 2 inflammation.

ILC2s play a pivotal role in parasitic immune responses and allergic reactions [3]. These cells drive type 2 innate immunity by producing key cytokines IL-4, IL-5 and IL-13 in response to stimulation by IL-25, IL-33, and thymic stromal lymphopoietin [28]. In our model, ILC2 frequencies were increased in the AR and IL-33+AR groups compared with controls, supporting their involvement in allergic inflammation. However, the current study did not provide evidence that nasal microbiota dysbiosis can further activate ILC2s in this context. Notably, we did not observe significant differences in the memory-like ILC2s (ICOS^+^ST2^+^ subset) subset among groups. Although ICOS^+^ST2^+^ ILC2s have been linked to memory-like recall responses in allergic airway models [16], this phenotype was not clearly enriched in our AR setting, possibly due to the low abundance of nasal ILC2s and context-dependent differences between AR and asthma. Therefore, ICOS^+^ST2^+^ ILC2s may primarily reflect alarmin responsiveness here, and ILC2 memory-like programming in AR requires further validation using functional and epigenetic assays.

MiR-155 plays an important role in both allergen-induced airway inflammation and the pathogenesis of Th2-mediated allergic responses [29,30].Meanwhile,MiR-155 helps regulate Th2 cytokine production in ILC2s and mediating allergic inflammatory responses in AR [8]. In our study, miR-155 expression was significantly higher in the IL-33+AR group compared to controls, while the antibiotic + AR group showed a non-significant increase. These results support a potential contribution of miR-155 to IL-33–augmented type 2 inflammation, although the mechanistic relationship between miR-155 and ILC2 function in nasal tissue warrants further study. MiR-155 has been reported to modulate epithelial barrier integrity by affecting tight junctions and epithelial permeability, contribute to tissue remodeling, reshape microbial community structure, and regulate inflammation-related signaling pathways [[31], [32], [33]]. Although our study did not establish a mechanistic link between miR-155 and microbiota alterations or autophagy/mitophagy, the observed changes in miR-155 expression support its potential involvement in the broader inflammatory network of AR and warrant further functional validation in future studies.

Increasing evidence links nasal microbiota composition with AR pathogenesis, although findings across cohorts remain inconsistent [6]. Che et al. proposed that nasal dysbiosis may contribute to the development of both AR and non-allergic rhinitis [34]. Paradoxically, some studies report AR patients have lower abundance and homogeneity of nasal microbiota [35]. In our study, both AR and antibiotic-treated AR mice showed reduced nasal α-diversity compared to controls, though not statistically significant. Interestingly, the IL-33+AR group showed significantly enhanced α-diversity and β-diversity compared to the AR group alone. Importantly, these observations indicate an association between IL-33 administration and shifts in nasal microbiota composition, but they do not establish causality regarding whether ILC2 activation drives the microbial changes or vice versa.

Nasal microbiota composition in AR remains controversial. At the phylum level, Proteobacteria, Firmicutes, and Actinobacteria were dominant across all groups. The IL-33+AR group showed decreased Proteobacteria and increased Firmicutes, with a modest increase in Actinobacteria. Prior studies have reported variable phylum-level changes in AR, including either increased Firmicutes or increased Proteobacteria depending on the cohort and sampling context [36,37]. Proteobacteria are mainly Gram-negative bacteria that contain lipopolysaccharide, which primarily suppresses the progression of allergic diseases through TLR-4 signaling. In our study, acinetobacter, a genus within Proteobacteria, showed significantly reduced abundance in the IL-33+AR group compared to the other three groups. Acinetobacter may inhibit allergic airway inflammation by inducing a Th1 immune response and suppressing the Th2 response [38,39]. The Firmicutes, as the core intestinal microbiota, ranked second in the abundance of nasal microbiota in all mouse groups, and its abundance was significantly increased in the IL-33+AR group. We speculate that the gut microbiota may influence the progression of AR by modulating the relative abundance of nasal microbiota. Staphylococcus, a genus within Firmicutes, was significantly elevated in the IL-33+AR group, showing statistical significance compared to the other groups. Consistent with previous studies, we hypothesize that Staphylococcus may contribute to AR pathogenesis by inducing nasal epithelial cells to release type 2 inflammatory mediators, thereby exacerbating Th2 cell–mediated inflammation in the nasal cavity [34,36,40]. Overall, our findings support an association between IL-33–augmented AR and nasal microbial dysbiosis, while acknowledging that mechanistic links remain to be tested.

Autophagy and mitophagy participate in chronic airway inflammation and allergic disease, but their roles in AR remain incompletely defined [22,41]. Some studies show increased autophagy-related proteins in AR [42], while others report impaired autophagy marked by lower LC3-II/I and Beclin1 and higher p62 after allergen exposure [43]. Our autophagy readouts showed increased p62 together with an elevated LC3-II/LC3-I ratio at the nasal mucosa level. These findings do not necessarily contradict each other, because LC3-II accumulation can reflect either enhanced autophagosome formation or reduced autophagosome clearance, whereas p62 elevation more consistently suggests impaired autophagic degradation/flux. Autophagy is a dynamic, multi-stage process. During the initiation phase, damaged organelles trigger the activation of Beclin-1, while LC3-II localizes to autophagosome membranes [44]. We hypothesize that this phenomenon may be associated with impaired mitophagy, leading to ROS accumulation [45], which subsequently damages lysosomal function and results in autophagosome accumulation. As we did not directly assess autophagic flux or measure oxidative stress, we interpret these results as indicative of dysregulated autophagy rather than definitively increased autophagy activity. Importantly, we observed decreased FUNDC1 and BNIP3L (NIX), two key receptors involved in receptor-mediated mitophagy and mitochondrial quality control at the nasal mucosa level. Downregulation of these pathways may favor the persistence of damaged mitochondria, which could increase susceptibility to oxidative stress and inflammatory signaling and thereby contribute to epithelial barrier dysfunction and tissue remodeling in AR. However, the functional consequences of FUNDC1/BNIP3L reduction and the potential involvement of ROS/lysosomal pathways remain to be experimentally validated in future studies.

ILC2s and autophagy both play roles in tissue remodeling in AR [5,22]. Activated ILC2s drive remodeling by producing IL-4 and IL-13, which act through the IL-4R/STAT6 pathway [46]. Autophagy serves as a critical regulatory factor in myofibroblast differentiation, tissue remodeling, and the progression of fibrosis [47]. In our study, mitophagy was significantly impaired in AR mice, possibly leading to damaged mitochondria, increased ROS, further compromise of the epithelial barrier function and promotion of fibrosis—contributing to tissue remodeling. It may also promote tissue remodeling by generating type 2 inflammatory factors through ILC2s.

This study has several limitations that should be acknowledged. First, our research was conducted using a mouse animal model, which may not fully recapitulate human disease pathophysiology. Secondly, autophagy results varied across AR groups, with some showing increased and others decreased activity. To clarify these findings, we propose employing more definitive approaches such as transmission electron microscopy for direct visualization of autophagosomes, or pharmacological modulation using autophagy-specific inhibitors/activators to systematically examine the functional contribution of autophagy in type 2 inflammation and tissue remodeling in AR. ILC2-specific autophagy requires future work. Another limitation of this study is that the in vivo experiments included only five mice per group, which may limit statistical power and generalizability; future studies with larger cohorts and independent validation are warranted. A major limitation is that an IL-33-alone group was not included; therefore, the independent contribution of IL-33 to type 2 cytokine induction cannot be fully separated from its combined effects with OVA challenge. Here, memory-like ILC2s are identified by phenotype: ICOS^+^ST2^+^ ILC2s, and further functional and epigenetic validation is required to confirm true ILC2 memory.

## 5 Conclusion

In summary, AR, IL-33+AR, and antibiotic + AR mouse groups were established and compared with controls. IL-33 co-treatment was associated with a more prominent type 2 inflammatory profile than AR alone. Histology suggested epithelial barrier disruption in AR-related groups. Nasal mucosal tissue remodeling is significantly enhanced in AR groups compared to controls, potentially associated with type 2 inflammation levels. We found no evidence supporting an increase in “memory-like” ILC2s using the current marker strategy. IL-33 administration effectively activates ILC2s and is associated with significant changes in the composition of the nasal microbiota, particularly manifesting as reduced Proteobacteria and increased Firmicutes abundance; At the phylum level, Proteobacteria, Firmicutes and Actinobacteria represent the dominant microbiota across all groups. Dysregulated autophagy and mitophagy likely participate in AR development.

## Author contributions

Chen Wang: Methodology, Data curation, Formal analysis; writing—original draft. Yi-Ming Zhang: Methodology, software. Min-Li Zhou: Data curation, software. Min Li: Formal analysis, Methodology. Ke‐Jia Cheng: Writing—review&; editing. All authors have read and approved the manuscript.

## Ethics statement

This study was approved by the Research Ethics Committee of the First Affiliated Hospital, College of Medicine, Zhejiang University (Zhejiang, China) (licence no:2019933). The mice were housed in a specific-pathogen-free isolation environment in the Animal Experimental Center of The First Affiliated Hospital of Medical School of Zhejiang University (licence no. SYXK(Zhe)2023-0021).

## Funding statement

This research were funded by the Youth Science Foundation Project of 10.13039/501100004731Zhejiang Provincial Natural Science Foundation (Grant No. LQ23H130001).

## Declaration of competing interest

The authors declare that they have no known competing financial interests or personal relationships that could have appeared to influence the work reported in this paper.

## Figures and Tables

**FigS1:**
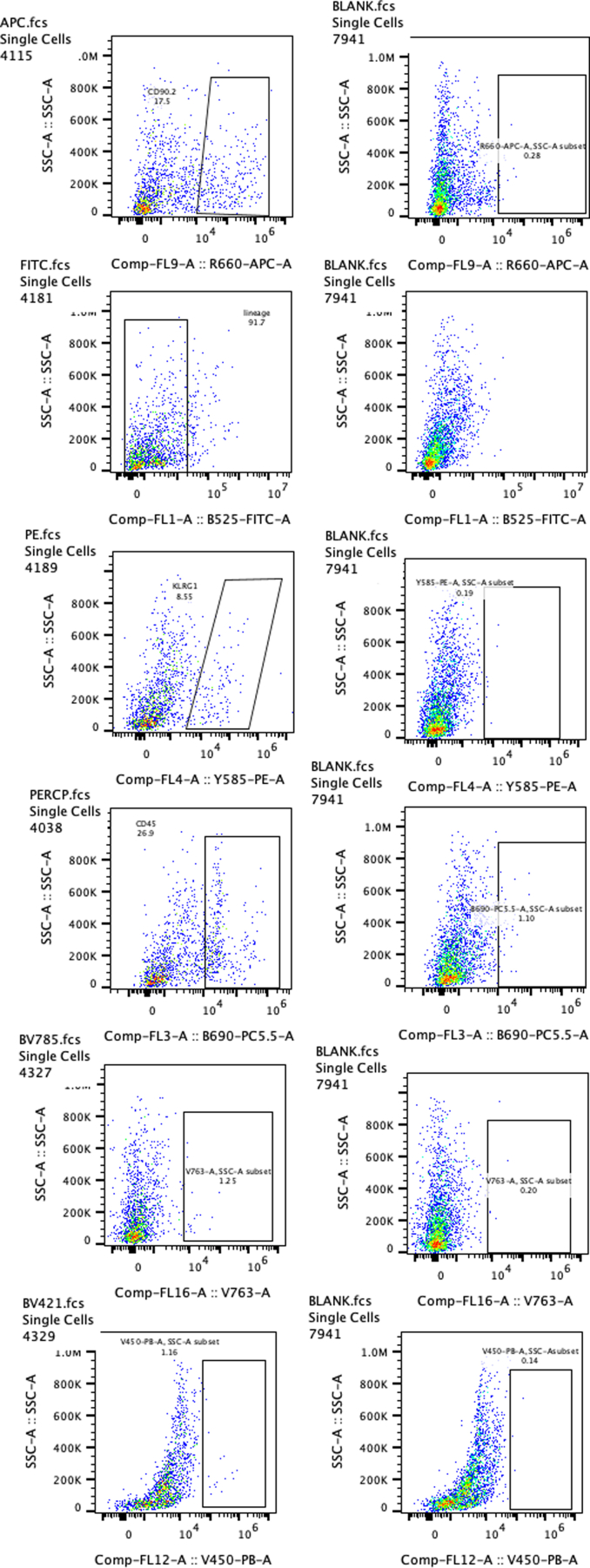


## Data Availability

Data will be made available on request.

## Abbreviations

AR: allergic rhinitis
ILC2s: Type 2 innate lymphoid cells
ILCs: innate lymphoid cells
Th: T helper
IL: interleukin
miR-155: microRNA-155
OVA: ovalbumin
PBS: phosphate buffered saline
HE: hematoxylin-eosinstaining
NLF: nasal lavage fluid
ELISA: enzyme-linked immunosorbent assays
DEPC: diethyl pyrocarbonate
16S rRNA: 16S Ribosomal RNA Gene Sequencing
PCoA: principal coordinate analysis
LC3: light chain 3
FUNDC1: FUN14 domain-containing protein 1
PINK1: phosphatase and tensin homolog-induced putative kinase 1
BNIP3: BCL2-interacting protein 3
