# Supplementary figures and images for: The role of nasal microbiota and type 2 innate lymphoid cells in the pathogenesis of allergic rhinitis
Source: Biochem Biophys Rep. 2026 May 4;46:102602. doi: 10.1016/j.bbrep.2026.102602 (PMC13158566; doi:10.1016/j.bbrep.2026.102602)

Fig6-A

P62


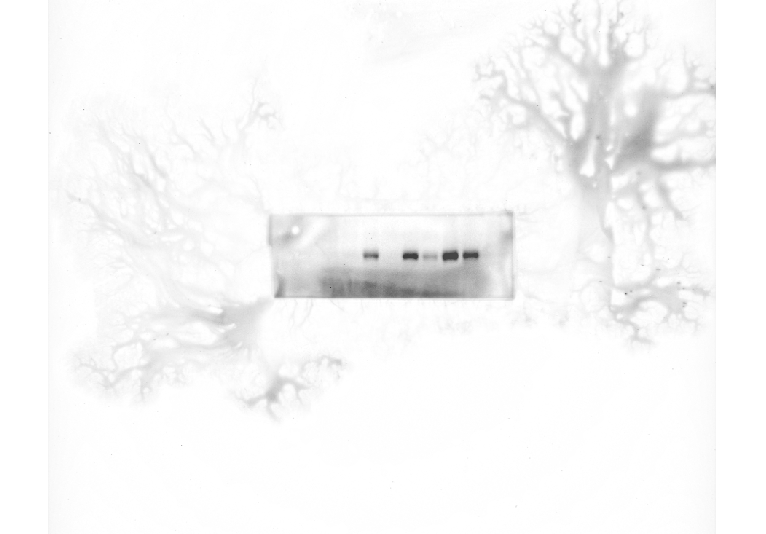


75kDa


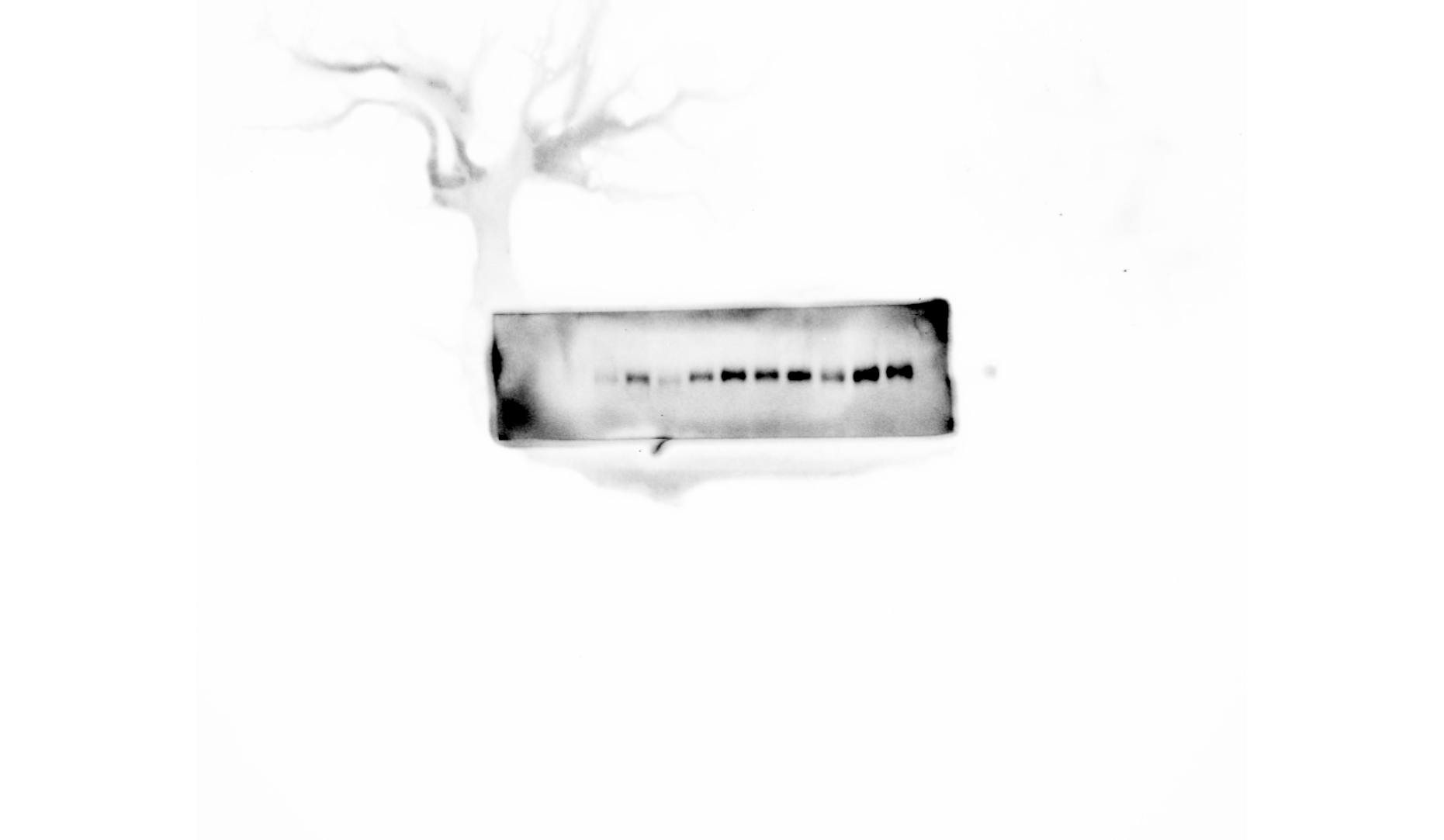


LC3

25kDa


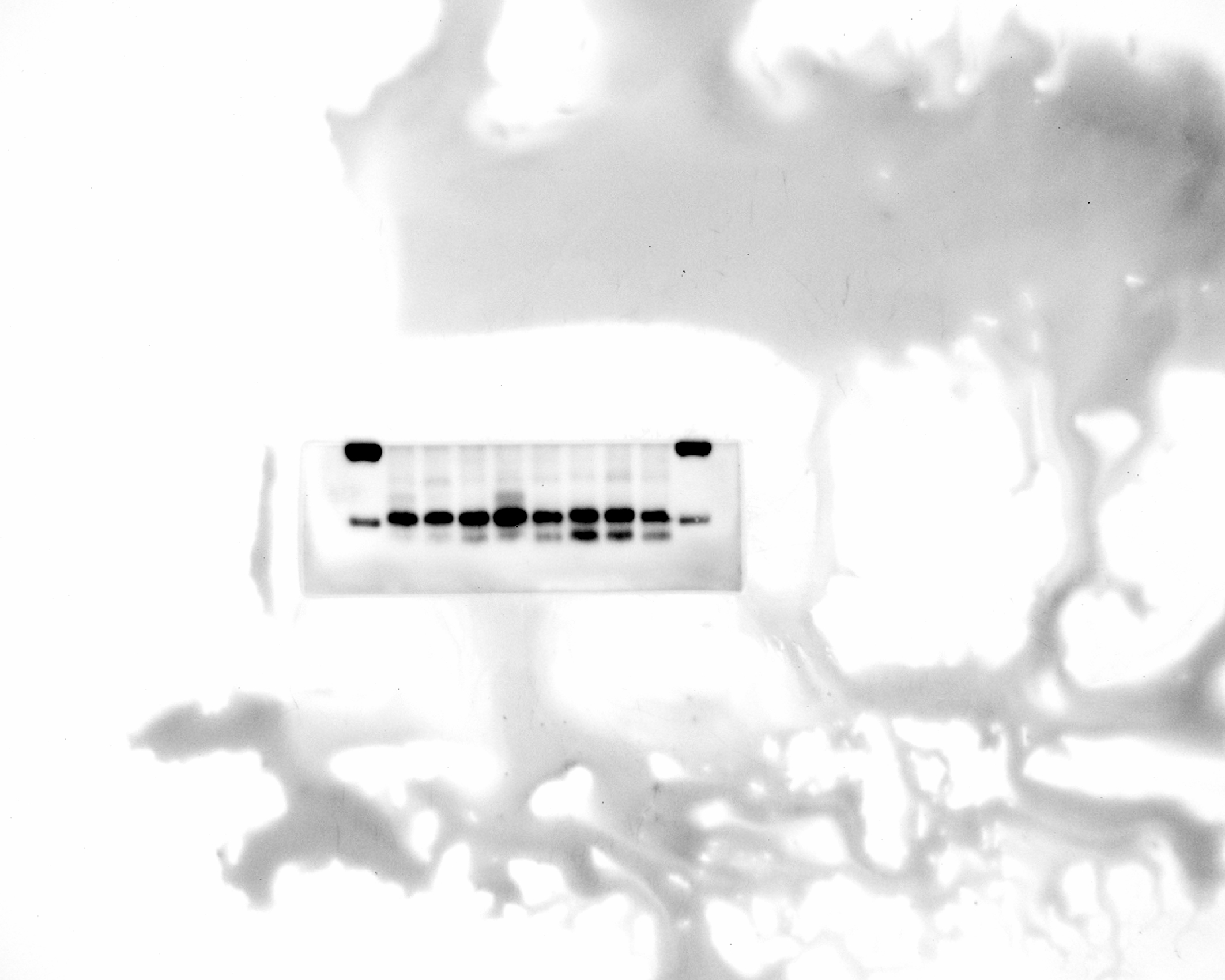


17kDa


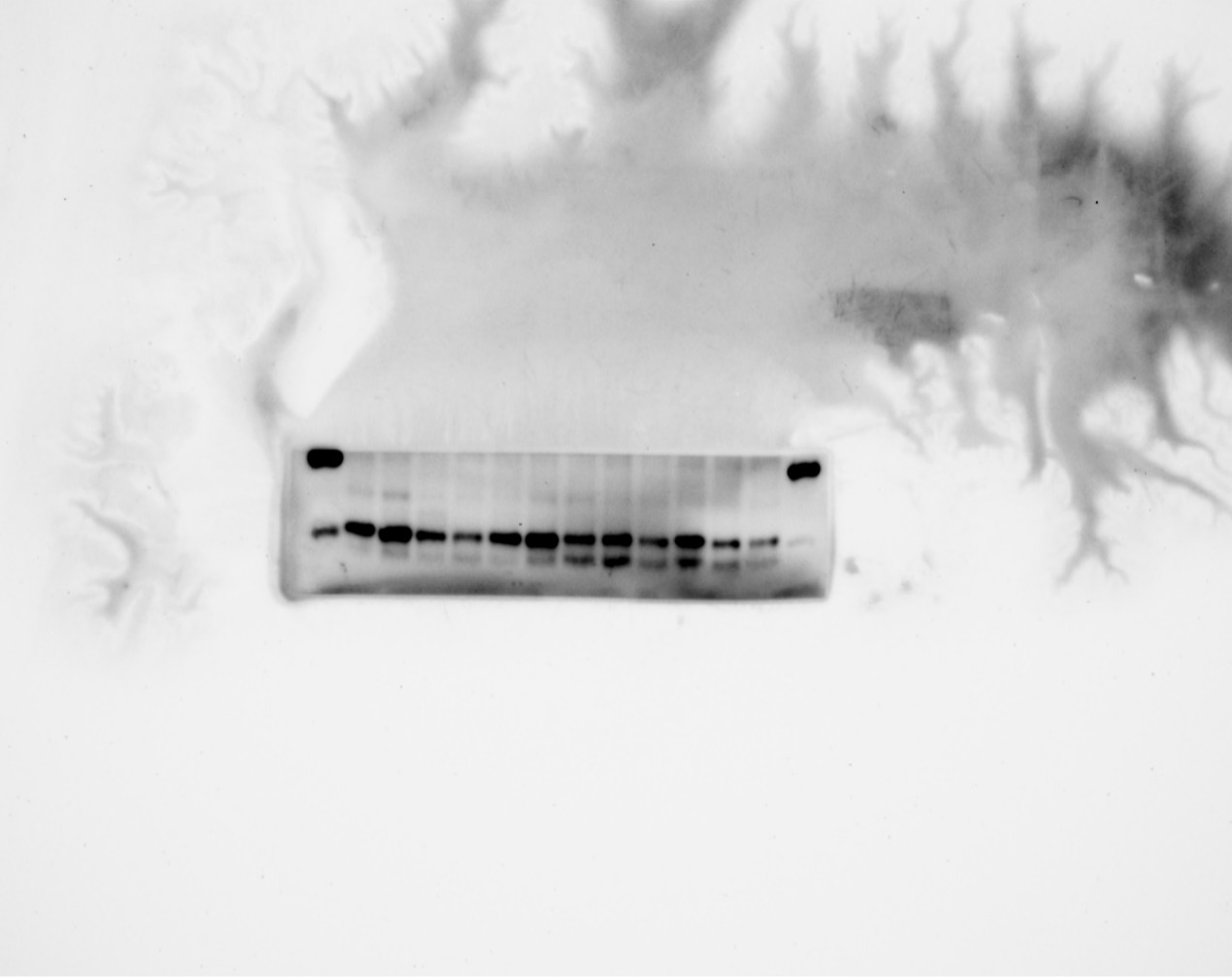


Beclin1

75kDa


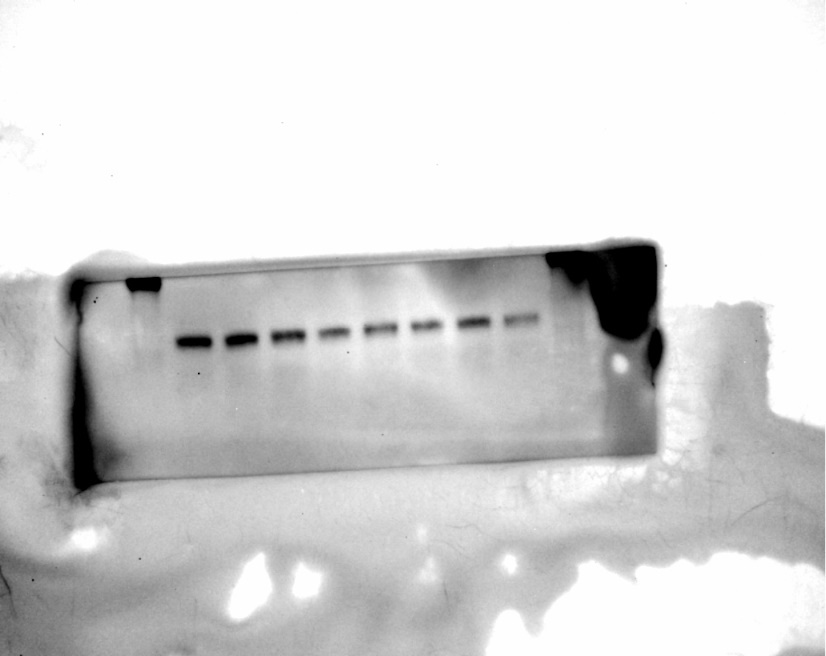


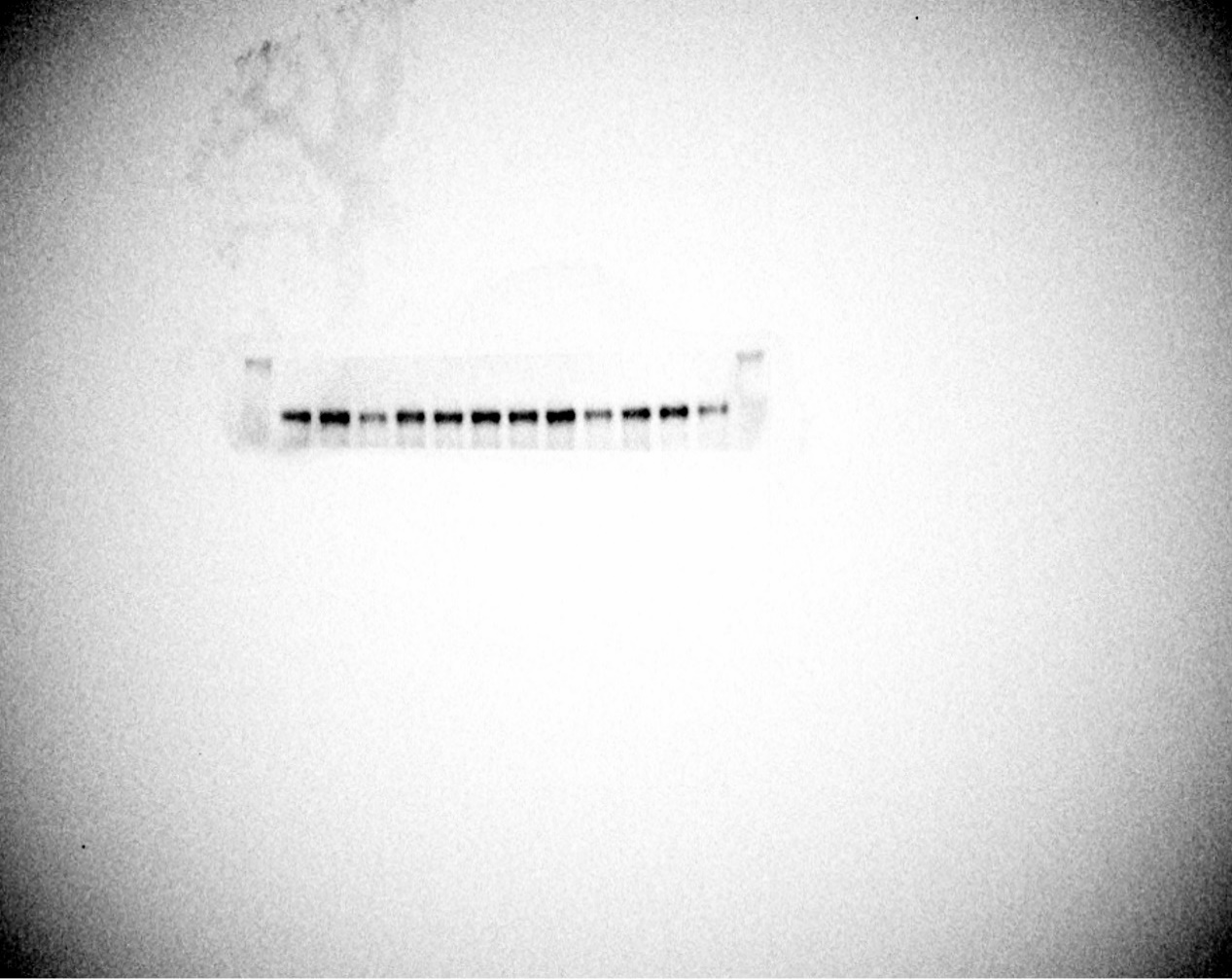


β-actin


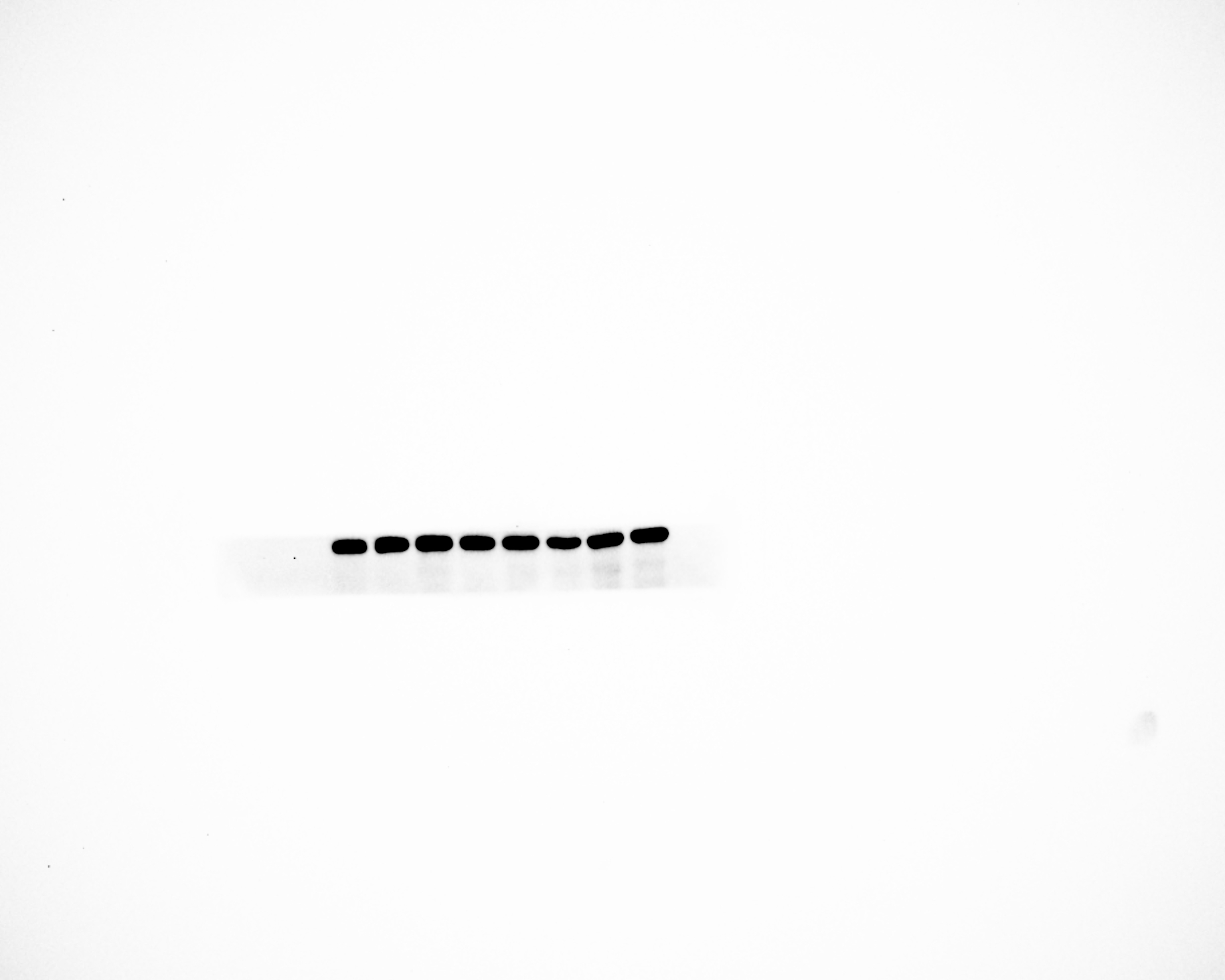


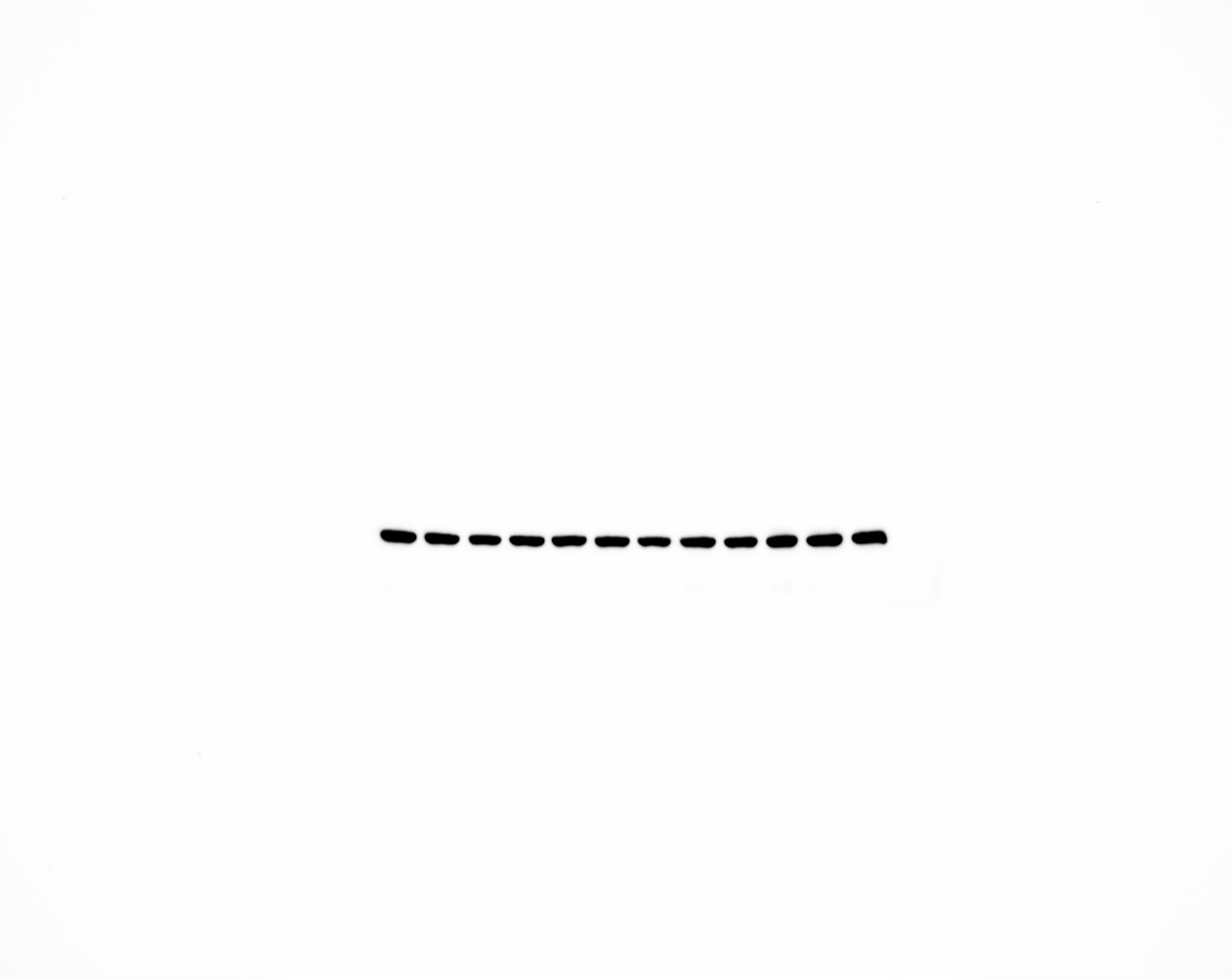


Fig6-E

Parkin

75kDa


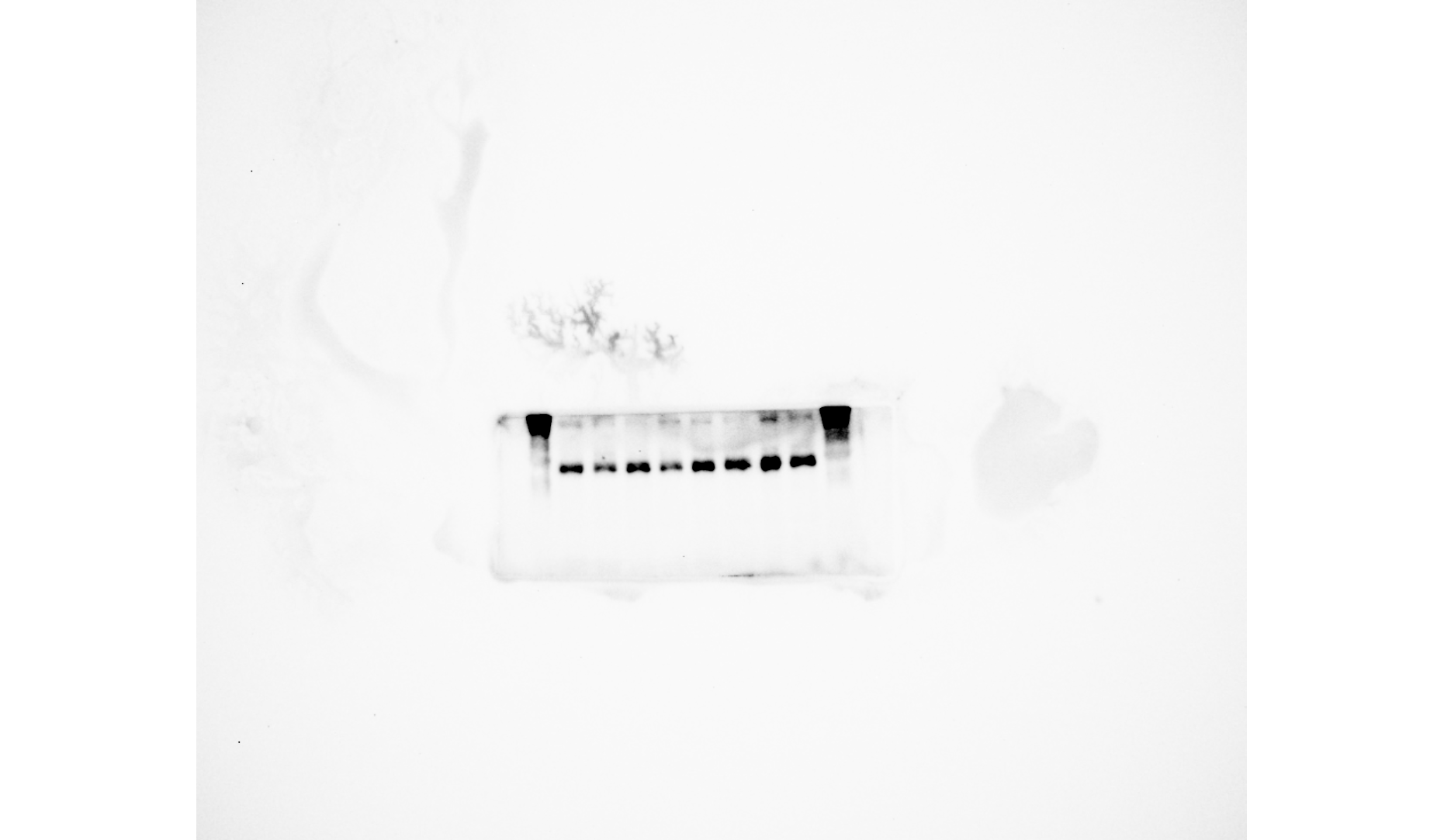


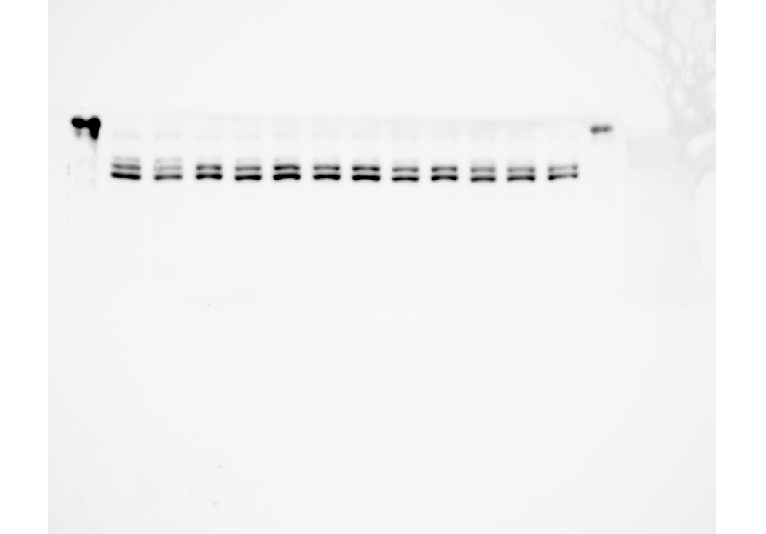


Pink1

75kDa


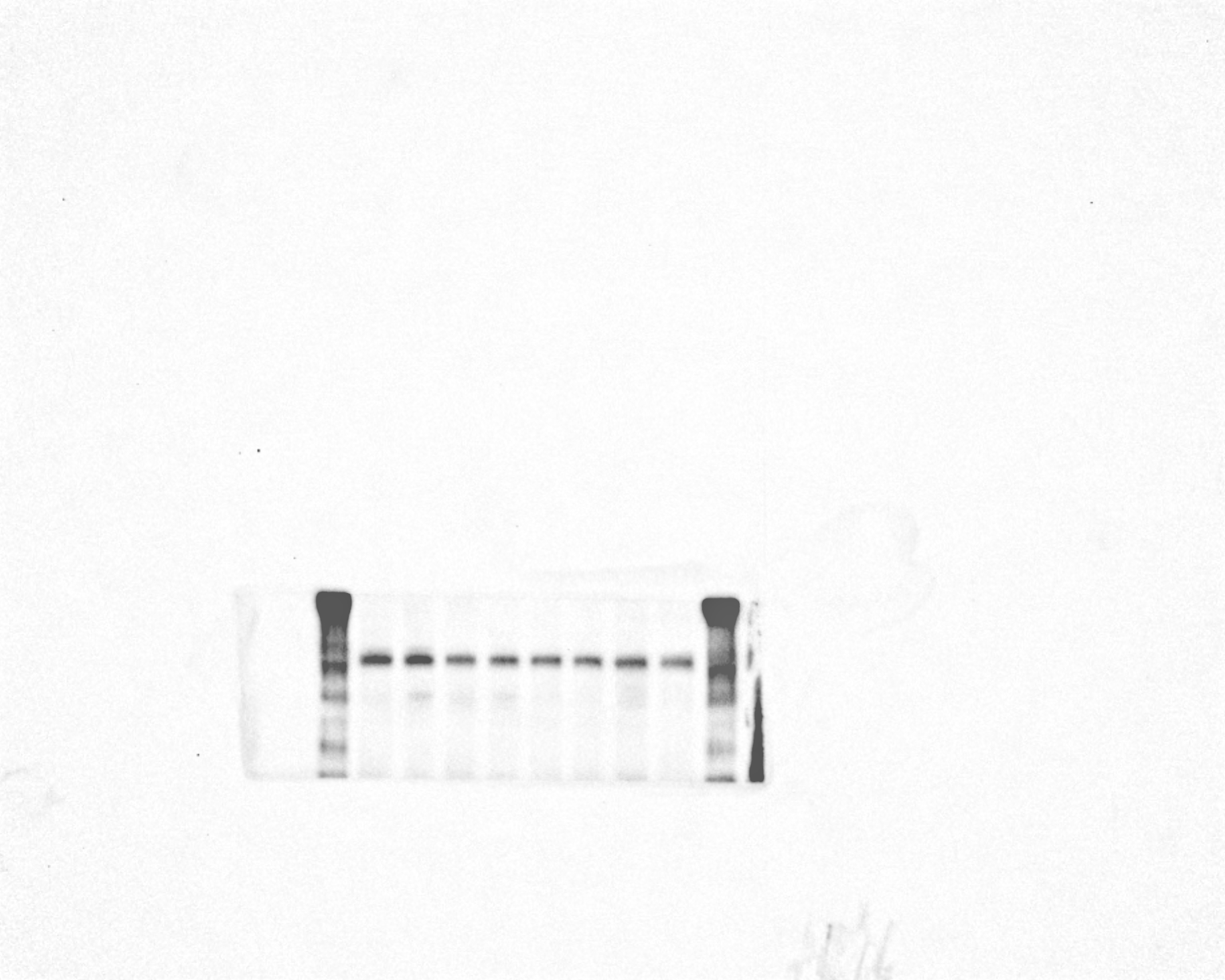


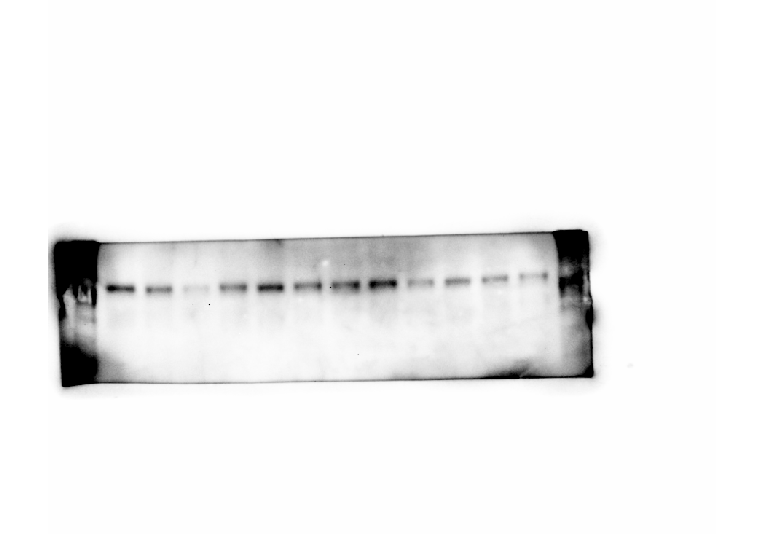


BNIP3L


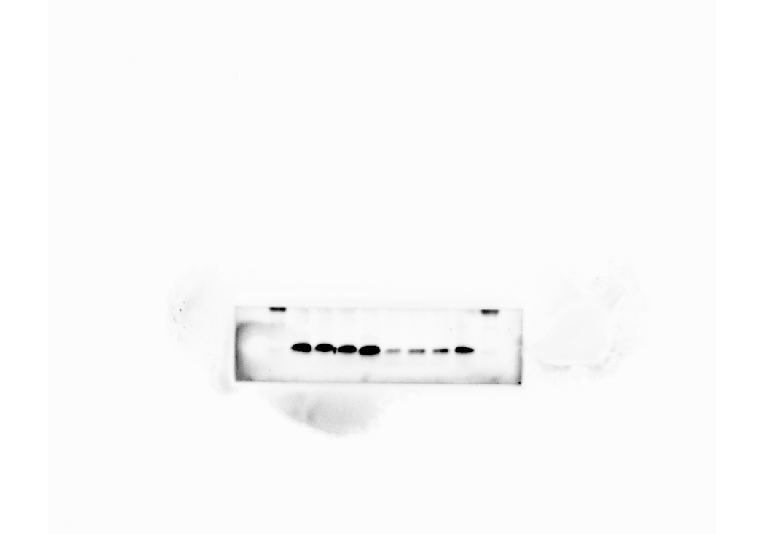


25kDa


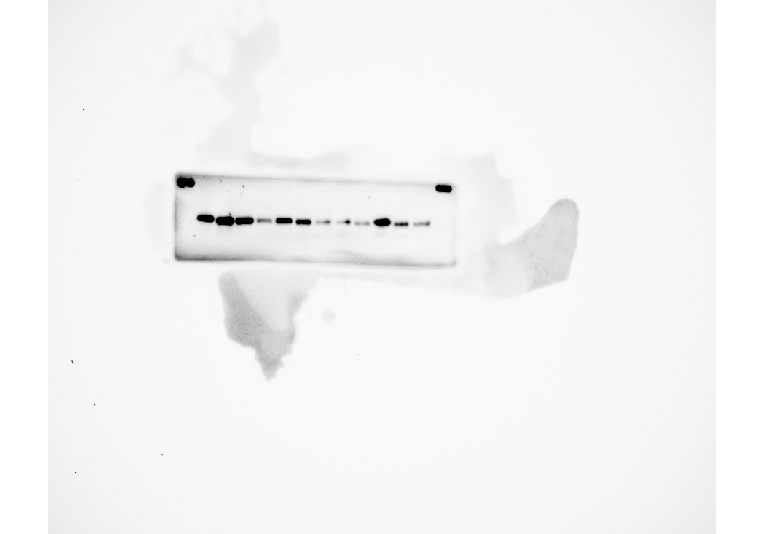


\

FUNDC1


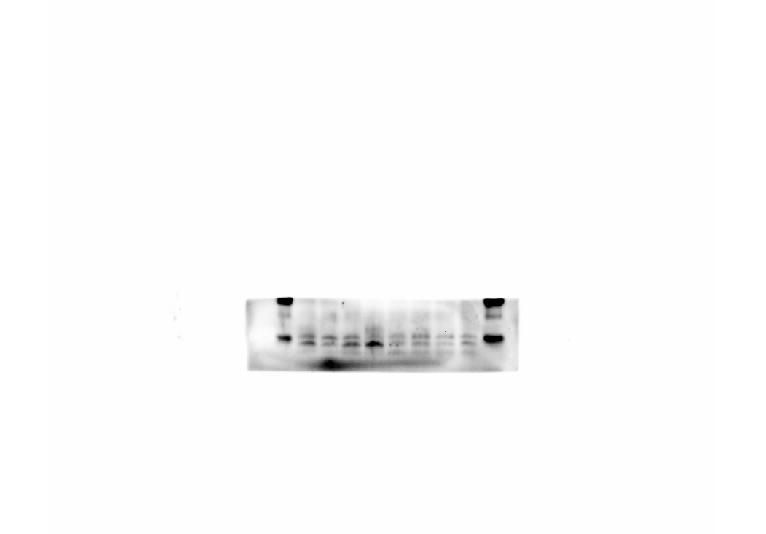


25kDa


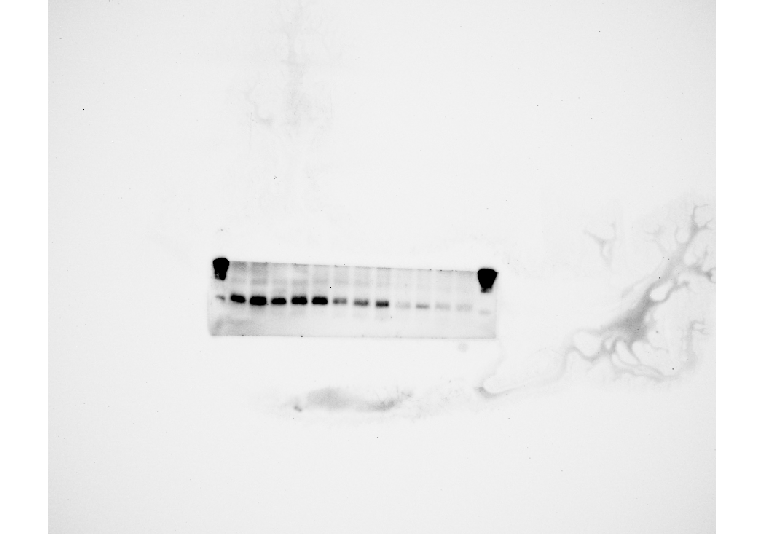


β-actin


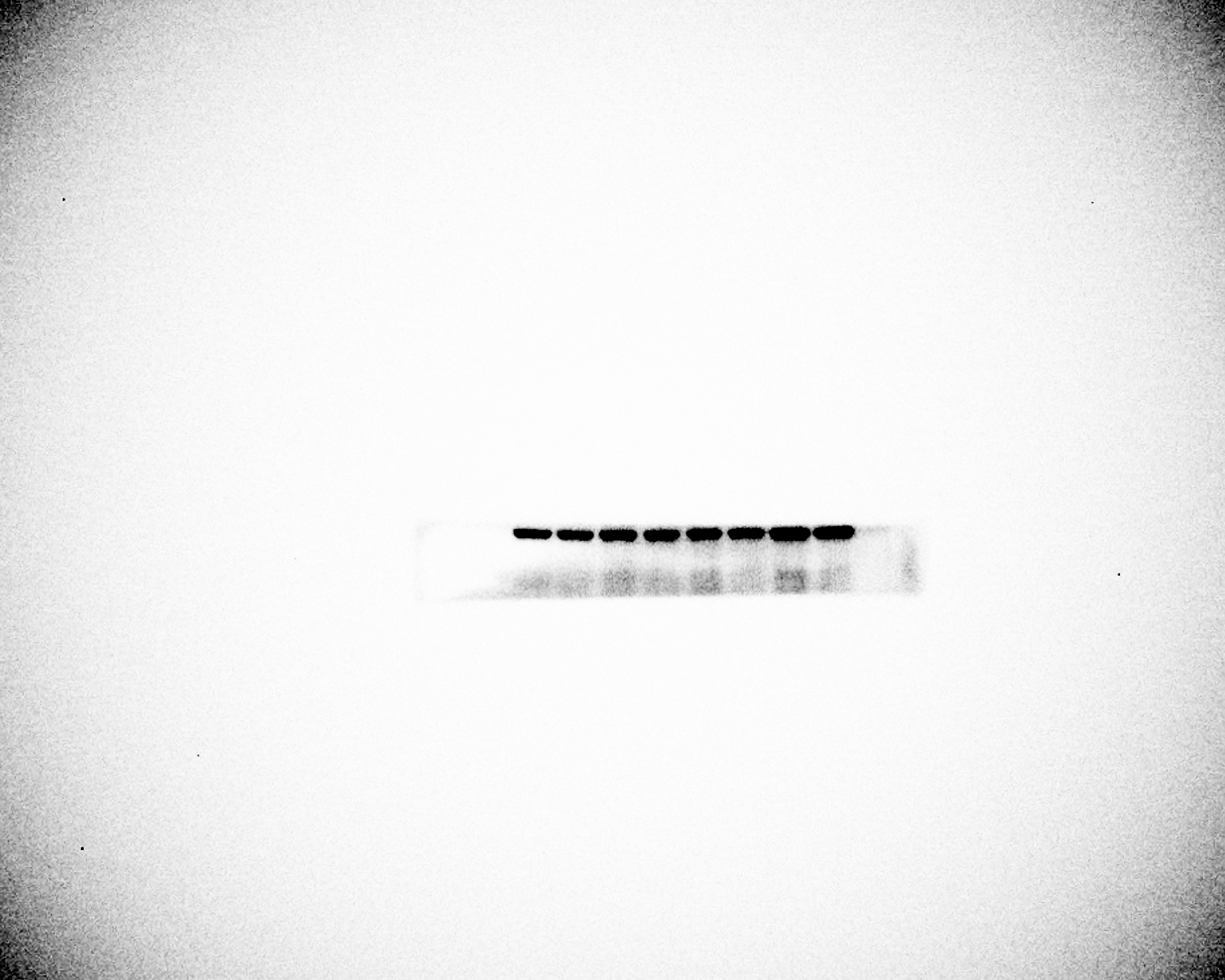


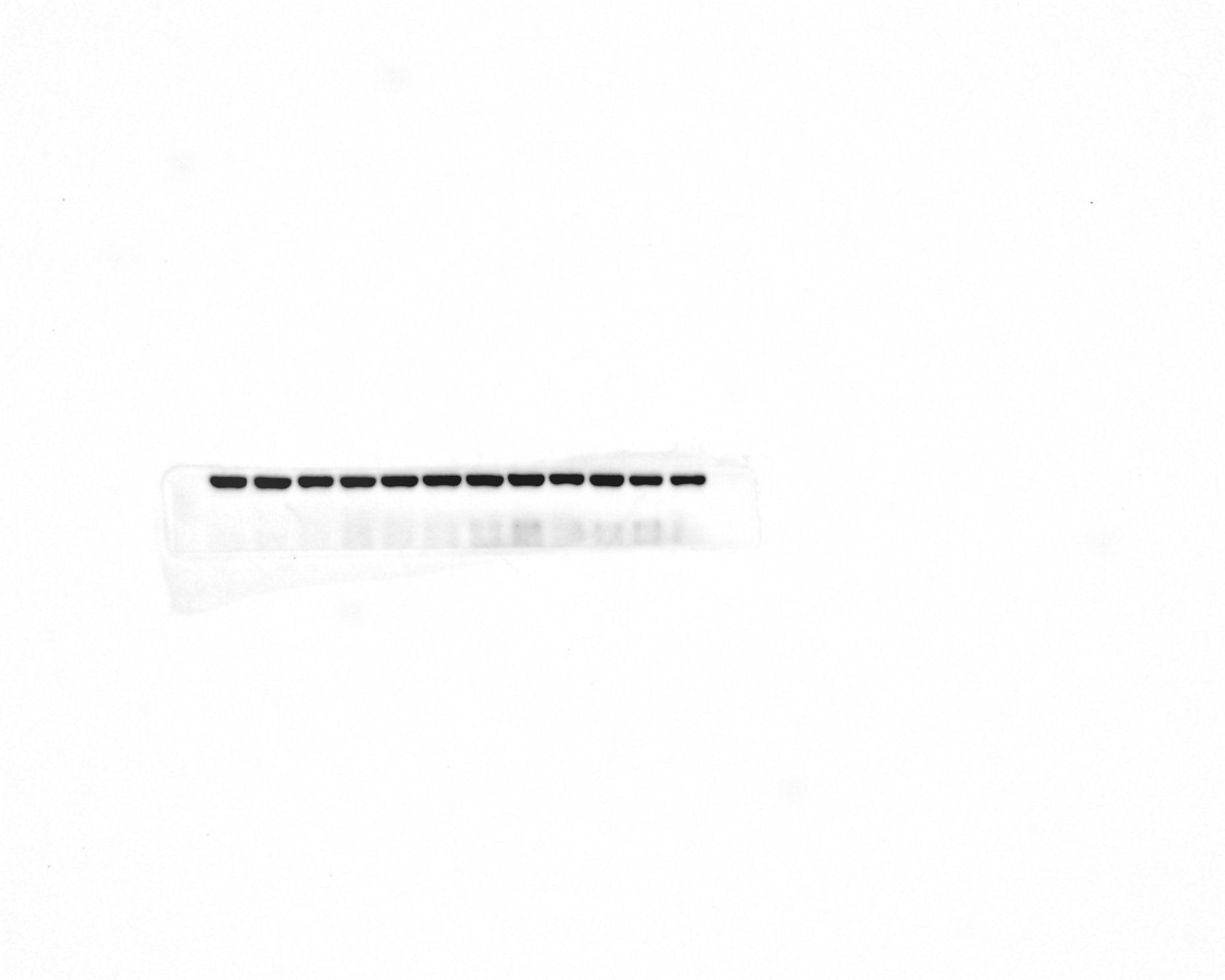

Supplement: Multimedia component 1 [file mmc1.docx]
